# Effects of Physical Activity on Disordered Eating Behaviours in Individuals With a Psychotic Disorder

**DOI:** 10.1111/eip.13611

**Published:** 2024-08-28

**Authors:** Kathya Carrier, Amal Abdel‐Baki, Lorilou Thériault, Antony D. Karelis, Tania Lecomte, Ahmed Jérôme Romain

**Affiliations:** ^1^ Faculty of Arts and Sciences, Department of Psychology University of Montreal Montreal Quebec Canada; ^2^ Faculty of Medicine, Department of Psychiatry and Addictology University of Montreal Montreal Quebec Canada; ^3^ Centre de Recherche du Centre Hospitalier de l'Université de Montréal Montreal Quebec Canada; ^4^ Department of Physical Activity Sciences University of Quebec in Montreal Montreal Quebec Canada; ^5^ Centre de Recherche de l'Institut Universitaire en Santé Mentale de Montréal Montreal Quebec Canada; ^6^ School of Kinesiology and Physical Activity Sciences, Faculty of Medicine University of Montreal Montreal Quebec Canada

**Keywords:** cognitive restraint, eating behaviour, emotional eating, physical activity, psychotic disorder, uncontrolled eating

## Abstract

**Aim:**

This study aims to determine the effect of physical activity on cognitive restraint, uncontrolled eating and emotional eating in individuals with a psychotic disorder.

**Methods:**

Twenty‐seven participants with a psychotic disorder (55% male; mean age: 30 ± 7.5 years; Caucasian: 66.7%; schizophrenia spectrum disorders: 44.4%; bipolar disorder with psychotic features: 29.6%) took part in a 6‐month bi‐weekly physical activity program (walking, running, yoga, cycling and dancing). The Three‐Factor Eating Questionnaire was used to assess participant's eating behaviours, and the frequency of completed physical activity sessions was compiled.

**Results:**

The mixed models analysis approach revealed that the level of cognitive restraint remained unchanged (pre: 39.2 ± 18.7 vs. post: 44.1 ± 18.3; *p* = 0.24), while the levels of uncontrolled eating (pre: 39.7 ± 19 vs. post: 31.6 ± 19.7; *p* = 0.02) and emotional eating (pre: 45.5 ± 22.3 vs. post: 32.2 ± 22.2; *p* < 0.001) decreased at the end of the 6‐month physical activity program.

**Discussion:**

This study showed that physical activity has positive effects on disordered eating behaviours in individuals with a psychotic disorder, similarly to previous studies on other populations (e.g., overweight and obese participants, postmenopausal women).

**Conclusion:**

Further studies are warranted to better understand the role of physical activity in moderating eating behaviours.

## 1 Introduction

Psychotic disorders are marked by symptoms like hallucinations and delusions, and include schizophrenia spectrum disorders like schizoaffective disorder, and mood disorders such as major depression and bipolar disorder with psychotic features. These disorders are associated with persistent challenges across cognitive, behavioural, psychosocial and physical health aspects of functioning (Kouidrat et al. 2014; Kouidrat, Amad, and De Hert 2015; Li et al. 2020; McIntyre et al. 2020).

Individuals with a psychotic disorder have an increased risk of developing a metabolic syndrome, characterized by elevated blood pressure, high glucose levels, large waist circumference and impaired lipid levels. The metabolic syndrome doubles the risk of premature morbidity and mortality from cardiovascular issues, reducing life expectancy in individuals with a psychotic disorder by up to 20 years (Alberti et al. 2009; Larsen et al. 2019). Lifestyle factors like physical inactivity and disordered eating behaviours play a significant role in increasing the risk for a metabolic syndrome (Jakobsen et al. 2018). Thirty percent to 41% of individuals with a psychotic disorder exhibit disordered eating behaviours including cognitive restraint (control over food intake to control weight), uncontrolled eating (overeating due to loss of control over ingestion or excessive hunger) and emotional eating (overeating in response to emotions) (Duarte, Palmeira, and Pinto‐Gouveia 2020; Khosravi 2020). These disordered eating behaviours are more present when compared to healthy control (Kouidrat et al. 2018) and tend to be more frequent among individuals not receiving optimal treatments, such as psychiatric rehabilitation or medication adjustments to manage adverse effects (Khosravi 2020).

To modify these disordered eating behaviours, physical activity has been considered as a potential intervention. Previous studies showed inverse association between physical activity and these disordered eating behaviours (Aymes et al. 2022; Romain et al. 2022). Furthermore, it has been documented that these disordered eating behaviours are associated with depressive symptoms, negative mood, low self‐efficacy, low self‐regulation, high body mass index and physical activity is known to have an impact on these factors (Annesi 2021; Annesi and Eberly 2022; Aymes et al. 2022; Costa et al. 2021; Dohle, Hartmann, and Keller 2014). Physical activity can decrease one's craving for food, help recognize natural triggers of thirst and hunger which can be deficient in uncontrolled eating, as well as calm anxiety or emotional distress that could lead to emotional eating (Malchow et al. 2013).

Physical activity also benefits people with a psychotic disorder by enhancing quality of life, cognition and mood (Malchow et al. 2013; Rosenbaum et al. 2014; Stubbs et al. 2018). It is a valuable adjunct therapy, yet research on its impact on disordered eating behaviours in individuals with a psychotic disorder is non‐existing. Since a quarter of individuals with a psychotic disorder experience metabolic syndrome and half exhibit disordered eating behaviours, addressing these issues is crucial (Carrà et al. 2014). Exploring the impact of physical activity, known to improve cognitive restraint, uncontrolled eating and emotional eating in the general population, on disordered eating behaviours among individuals with a psychotic disorder is necessary (Bryant et al. 2012; Martinez‐Avila et al. 2020; Mason et al. 2019; Svensson et al. 2014).

The present study aimed to determine the effects of physical activity on cognitive restraint, uncontrolled eating and emotional eating in individuals with a psychotic disorder undertaking a supervised physical activity program. We hypothesized that physical activity would increase cognitive restraint and decrease uncontrolled eating and emotional eating.

## 2 Methods

This study is a comparative pre–post intervention study design. The data stem from two studies focusing on the impact of a bi‐weekly supervised physical activity intervention for individuals with a psychotic disorder (Dubois 2020; Romain et al. 2019). The first was a randomized controlled study with an intervention group (Romain et al. 2019) and the second was an open study (Dubois 2020). Between the 2 studies, a total sample size of 56 participants were included in the intervention groups; however, only 27 participants completed all the pre‐ and post‐intervention assessments (Dubois 2020) and were considered for the present study (*n* = 27).

The hypothesis and analysis plan were specified before the data were collected. Both studies received approval from the Ethics Committee of the research centre at the Centre Hospitalier de l'Université de Montréal (#10.255 and 16.399, respectively). All participants provided written informed consent.

### 2.1 Participants

Participants diagnosed with a psychotic disorder based on the Diagnostic and Statistical Manual of Mental Disorders, Fourth Edition, Text Revision (DSM‐IV‐TR) were recruited from 2012 to 2018 through the early intervention for psychosis program, outpatient psychotic disorders program and affective disorders program of the Department of Psychiatry at the Centre Hospitalier de l'Université de Montréal (Montreal, Canada).

#### 2.1.1 Inclusion Criteria

To be included, potential participants (1) had to be 18–55 years old; (2) have a diagnosis of schizophrenia, schizoaffective disorder, bipolar disorder or major depressive disorder with psychotic features; (3) had no or stable psychotic symptoms for at least 1 month; (4) take medication at therapeutic doses for which there was no plan of changing it for the next 6 months; (5) had to be overweight or obese (BMI ≥25 kg/m^2^); (6) have an abdominal circumference meeting the criteria for metabolic syndrome (≥94 cm for men, ≥80 cm for women) (Goldenberg and Punthakee 2013); (7) had to be inactive, that is, engaging in less than two sessions of structured physical activity per week (Romain et al. 2019), or practicing physical activity less than 90 min per week (Dubois 2020).

Potential participants were not eligible if they had a medical contraindication to exercise or an orthopaedic limitation. In Romain et al.'s (2019) study, potential participants were not eligible if they (1) had an uncontrolled cardiovascular disease, (2) had Type 1 or 2 diabetes, (3) had a diagnosed eating disorder, (4) had an uncontrolled thyroid and pituitary gland disorder, (5) had pregnancy or were planning to become pregnant in the next year, (6) had an excessive alcohol consumption (>2 drinks per day), (7) were using depot medroxyprogesterone acetate as a contraceptive method, (8) were in menopause or in perimenopause, (9) were using medication for comorbid medical conditions that could affect the results (e.g., metformin), except for psychotropic medication, would prevent the participants from participating.

### 2.2 Intervention

In Romain et al.'s (2019) study, participants in the intervention group underwent bi‐weekly 30‐min high‐intensity interval training sessions on a treadmill for 6 months, including warm‐up and rest periods. In Dubois' (2020) study, the intervention involved bi‐weekly 45‐min sessions over 6 months. The first 3 months comprised supervised activities based on participant preferences (walking, running, yoga, cycling and dancing), with behaviour change techniques applied by a kinesiologist supervisor.

The current study combined samples to enhance statistical power. Conducted by the same investigators in a single location, both studies involved similar individuals and their secondary aim was to examine disordered eating behaviours. Each study implemented a 6‐month bi‐weekly physical activity program tailored to individuals with a psychotic disorder, collecting comparable eating behaviour data. Hypotheses and analyses were predetermined prior to data collection.

### 2.3 Data Collection

Demographic information as well as the diagnosis were gathered from the participants' medical records and from treating psychiatrists. Data on eating behaviours were collected pre‐intervention (T1) and post‐intervention, that is, after 6 months of intervention (T2).

### 2.4 Measures

The frequency of participation in physical activity sessions and sociodemographic information were recorded during research interviews conducted by the researchers. Depending on the study, two versions of the Three‐Factor Eating Questionnaire (TFEQ‐51 and TFEQ‐R21) were used to assess eating behaviours at T1 and T2.

The TFEQ‐51 (Stunkard and Messick 1985) is subdivided into 3 factors that is, cognitive restraint (21 questions), disinhibition (16 questions) and susceptibility to hunger (14 questions), meaning susceptibility to internal and external hunger signals (Löffler et al. 2015). The TFEQ‐51 demonstrates adequate internal consistency and good convergent and discriminant validity (Bohrer, Forbush, and Hunt 2015). This version was used by Bryant et al. (2012).

The TFEQ‐R21 (Cappelleri et al. 2009) is a revised, shorter version with 21 questions grouped into 3 factors: cognitive restraint (6 questions), uncontrolled eating (9 questions) and emotional eating (6 questions). It shows strong internal reliability and adequate convergent and divergent validity (Duarte, Palmeira, and Pinto‐Gouveia 2020). Kouidrat et al. (2018) used this version. For our research, we retained only the scores for Items 2, 4 and 10 from the TFEQ‐R21 as Items 7, 14 and 16 were absent in the TFEQ‐51 and were added to the TFEQ‐R21 (Tholin et al. 2005).

Each item retained from TFEQ‐R21 version is scored on a Likert scale, that is, 4‐ or 8‐point Likert scale. Each item of the TFEQ‐R21 varies between 1 and 4 points. Each question of the TFEQ‐51 is scored on a Likert scale or answered as true or false (Stunkard and Messick 1985) and is worth 1 point. For the three factors of the TFEQs, a raw score is calculated by adding up every points. To harmonize the two versions of the TFEQ, the raw scores were transformed to a 0–100 scale with this formula [((raw score—lowest possible raw score)/possible raw score range) * 100]. The frequently employed ‘half‐scale’ technique is used to make up for missing data on certain items. For more details about the items used and the harmonization of the two questionnaires, refer to Appendix.

## 3 Analyses

Repeated‐measures linear mixed model analyses were conducted using R Studio (version 2023.09.01 + 494). The package ‘lmer’ (Kuznetsova, Brockhoff, and Christensen 2017) and ‘effectsize’ (Ben‐Shachar, Lüdecke, and Makowski 2020) were used in R Studio to run respectively the repeated‐measures linear mixed models and determine the effect sizes. The effect size was calculated by doing a partial eta square $\left(\eta_{p}^{2}\right)$ based on an analysis of variance (ANOVA) and by convention, values of $\eta_{p}^{2}=0.01$, 0.06 and 0.14 represent small, medium and large effect size, respectively. A statistically significant effect corresponds to a *p* ≤ 0.05 (Richardson 2011).

### 3.1 Sensitivity Analysis

A sensitivity analysis was conducted to assess if individuals completing 64% or more of physical activity sessions exhibited improved outcomes on cognitive restraint, uncontrolled eating and emotional eating. The 64% cut‐off was based on a pilot study (Abdel‐Baki et al. 2013). Participants meeting this cut‐off (*n* = 19) were included for analysis.

A secondary sensitivity analysis was performed using imputed data to address potential bias related to missing data. We used the ‘mice’ package (van Buuren and Groothuis‐Oudshoorn 2011) to perform multiple imputation and replace missing data. Missing data accounted for 51.8% in cognitive restraint, uncontrolled eating and emotional eating. Data for participants missing at T2 were imputed, increasing the total from 27 to 56 participants after imputation.

## 4 Results

### 4.1 Demographic Information, TFEQ Results and Physical Activity Frequency

An average of 81.2% (SD = 26.8%) of physical activity sessions were completed by the participants. Participants' characteristics (demographic and clinical data) are in Table 1 and eating behaviours (TFEQ) in Table 2.

**TABLE 1:** Participants' characteristics (*n* = 27).

Demographics	
Age (years) (*M* ± SD)	30 (7.5)
Sex, *n* (%)	
Male	15 (55.6%)
Female	12 (44.4%)
Ethnicity, *n* (%)	
African American or Afro‐Caribbean	3 (11.1%)
Asian	1 (3.7%)
Caucasian	18 (66.7%)
Latino/Latina American	2 (7.4%)
Other	3 (11.1%)
Relationship, *n* (%)	
Single	20 (74.1%)
Married or cohabitation	7 (25.9%)
Highest completed diploma, *n* (%)	
Elementary school	2 (7.4%)
High school	9 (33.4%)
College	7 (25.9%)
Professional studies diploma	1 (3.7%)
University	8 (29.6%)
Diagnostic, *n* (%)	
Bipolar disorder with psychotic features	8 (29.7%)
Schizophrenia	5 (18.5%)
Schizoaffective disorder	7 (25.9%)
Psychosis NOS	4 (14.8%)
Major depressive disorder with psychotic features	3 (11.1%)

*Note: M* ± SD = mean ± standard deviation, *n* (%) = number of participants (percentage).

Abbreviation: NOS = not otherwise specified.

**TABLE 2:** Eating behaviours mean scores, *p* value and effect size.

Eating behaviours	Time 1 (*M* ± SD)	Time 2 (*M* ± SD)	Change in time △ (*p* value)	Effect size ($\eta_{p}^{2}$)
Cognitive restraint				
Total sample (*n* = 27)	39.2 (18.7)	44.1 (18.3)	4.82 (0.24)	0.05
≥64% sessions	40.85 (18.46)	44.36 (16.58)	4.82 (0.24)	0.05
Total sample imputed data (*N* = 56)	36.78 (19.06)	45.98 (18.18)	9.20 (0.006*)	0.13
≥64% sessions (imputed data)	38.74 (18.25)	44.59 (17.04)	9.20 (0.006*)	0.13
Uncontrolled eating				
Total sample (*n* = 27)	39.7 (19)	31.6 (19.7)	−8.09 (0.02*)	0.20
≥64% sessions	36.84 (18.27)	25.99 (16.04)	−8.09 (0.02*)	0.20
Total sample imputed data (*N* = 56)	40.46 (22.95)	28.73 (16.32)	−11.73 (<0.001*)	0.22
≥64% sessions (imputed data)	38.64 (19.64)	26.70 (16.39)	−11.73 (<0.001*)	0.22
Emotional eating				
Total sample (*n* = 27)	45.5 (22.3)	32.2 (22.2)	−13.35 (<0.001*)	0.37
≥64% sessions	41.73 (22.37)	30.08 (22.15)	−13.35 (<0.001*)	0.37
Total sample imputed data (*N* = 56)	43.89 (25.46)	26.30 (19.76)	−17.59 (<0.001*)	0.42
≥64% sessions (imputed data)	42.21 (21.92)	28.90 (21.76)	−17.59 (<0.001*)	0.42

*Note: M* ± SD = mean ± standard deviation; $\eta_{p}^{2}=\text{partial}\;\mathrm{eta}\;\text{square}$.

* *p* ≤ 0.05.

### 4.2 Effect of Physical Activity on Disordered Eating Behaviours

We observed a significant decrease of the mean score change in the TFEQ for uncontrolled eating of 8.09 (*p* = 0.02; $\eta_{p}^{2}=0.2$) and a significant decrease of the mean score change in the TFEQ for emotional eating of 13.35 (*p* < 0.001; $\eta_{p}^{2}=0.37$).

Otherwise, we did not observe a statistical change in cognitive restraint (4.82; *p* = 0.24; $\eta_{p}^{2}=0.05$).

Regarding the association between completed physical activity session and disordered eating, we did not observe any significant association (cognitive restraint: *p* = 0.45, $\eta_{p}^{2}=0.02$; uncontrolled eating: *p* = 0.09, $\eta_{p}^{2}=0.11$; emotional eating: *p* = 0.35, $\eta_{p}^{2}=0.04$).

#### 4.2.1 Sensitivity Analysis: 64% or More of Physical Activity Sessions Completed

We observed a significant decrease of the mean score change in the TFEQ for uncontrolled eating of 8.09 (*p* = 0.02; $\eta_{p}^{2}=0.2$) when a participant completed 64% or more of the physical activity sessions. For every 1% increase in completed physical activity sessions, we observed a significant decrease of the mean score change in the TFEQ in uncontrolled eating of 14.24 (*p* = 0.051; $\eta_{p}^{2}=0.14$) when a participant completed 64% or more of the physical activity sessions. We observed a significant decrease of the mean score change in the TFEQ for emotional eating of 13.35 (*p* < 0.001; $\eta_{p}^{2}=0.37$) when a participant completed 64% or more of the physical activity sessions.

Otherwise, no statistical changes were found for cognitive restraint (4.82; *p* = 0.24; $\eta_{p}^{2}=0.05$).

Regarding the association between completed physical activity session and disordered eating behaviours, no associations were detected for cognitive restraint (3.22, *p* = 0.63; $\eta_{p}^{2}=0.01$) and emotional eating (9.98, *p* = 0.25; $\eta_{p}^{2}=0.05$).

#### 4.2.2 Sensitivity Analysis: Imputed Data

We found similar results for uncontrolled eating and emotional eating scores; however, a difference was observed on cognitive restraint (Table 2).

We found a significant increase of the mean score change in the TFEQ of 9.2 (*p* = 0.006; $\eta_{p}^{2}=0.13$) in cognitive restraint due to physical activity. Each 1% increase in completed sessions showed a non‐significant increase of the mean score change in the TFEQ of 0.02 in cognitive restraint (*p* = 0.7; $\eta_{p}^{2}=0.003$). Participants completing 64% or more sessions showed a significant increase of the mean score change in the TFEQ of 9.2 in cognitive restraint (*p* = 0.006; $\eta_{p}^{2}=0.13$), with each 1% session increase indicating a non‐significant increase of the mean score change in the TFEQ of 0.48 in cognitive restraint (*p* = 0.9; $\eta_{p}^{2}<0.001$). Physical activity explained 89.53% of cognitive restraint variance. With imputed data and a larger sample, physical activity demonstrated a medium to large effect on cognitive restraint.

## 5 Discussion

The present study aimed to determine the effects of physical activity on disordered eating behaviours among individuals with a psychotic disorder. As anticipated, the results on uncontrolled eating and emotional eating decreased significantly, regardless of whether the participants reached the completion threshold (more than 64% of physical activity sessions completed) set for the physical activity program. However, contrarily to our first hypothesis, no impact of physical activity on cognitive restraint was observed.

Our findings contrast with previous studies showing a notable increase in cognitive restraint post‐intervention (Bryant et al. 2012; Mason et al. 2019; Svensson et al. 2014). Two of these studies had higher weekly physical activity levels. It is plausible that cognitive restraint changes only after more exercise sessions. Additionally, all studies focused on weight loss, combining exercise with prescribed diets, potentially prompting participants to intensify food restrictions to manage weight, thereby increasing cognitive restraint. Our interventions focused solely on physical activity, excluding any nutritional or dietary components, with participants instructed to maintain their eating habits. Additionally, we did not differentiate between flexible cognitive restraint (permitting occasional deviations from a diet) and rigid cognitive restraint (strict adherence to diet rules) (Berg et al. 2018), potentially impacting our results. Flexible cognitive restraint is associated with weight loss and maintenance, whereas rigid cognitive restraint is linked to disinhibition and weight gain (Berg et al. 2018). Thus, not distinguishing between the two may obscure whether rigid or flexible cognitive restraint independently affects outcomes.

Our second hypothesis concerning the decrease of uncontrolled eating was validated by our results. Each 1% increase in physical activity leads to a decrease of 0.22 in uncontrolled eating. Over 6 months, regardless of program completion, uncontrolled eating significantly decreased by 8.09. Physical activity impact on uncontrolled eating aligns with prior studies (Bryant et al. 2012; Mason et al. 2019; Svensson et al. 2014). This effect may stem from physical activity's ability to enhance hunger and satiety signals awareness, potentially mitigating adverse effects of antipsychotic medications on appetite regulation (Carraça, Rodrigues, and Teixeira 2020). After exercise, the increase of leptin, a regulator of hunger and satiety, decreases food intake which may explain the decrease in uncontrolled eating (Lerma‐Cabrera, Carvajal, and Lopez‐Legarrea 2016).

Our third hypothesis regarding the decrease in emotional eating after physical activity was confirmed. Emotional eating significantly reduced by 0.14 with each 1% increase in physical activity session and by 13.35 after 6 months of activity, whether the program was completed, with a large effect size ($\eta_{p}^{2}=0.37$). Consistent with prior research by Mason et al. (2019) and Svensson et al. (2014), emotional eating declined after 3 and 12 months respectively. Our findings, along with those of Mason's and Svensson's, suggest that engaging in physical activity improves emotional eating regardless of the duration of activity involvement. Physical activity has a positive effect on mood symptoms by increasing serotonin which moderate appetite (Malchow et al. 2013; van Strien 2018).

According to Bruch's theory (Skårderud 2009), emotional eaters struggle to differentiate hunger from other emotional states, leading them to eat when anxious rather than when hungry. Physical activity may aid in distinguishing hunger from emotional cues, potentially reducing emotional eating (Dohle, Hartmann, and Keller 2014). Additionally, studies suggest physical activity may lessen emotional distress in individuals with a psychotic disorder, enhance mood, alleviate symptoms of anxiety and depression, and improve emotional self‐regulation, potentially mitigating emotional eating through its possible antidepressant effect via the serotonergic receptors 5‐HT1A (Dohle, Hartmann, and Keller 2014; Malchow et al. 2013).

This study explores the impact of physical activity on disordered eating behaviours in individuals with a psychotic disorder, pioneering a novel intervention approach. Its strengths include being among the first in this field and showcasing the potential effect of physical activity by not altering participants' eating behaviours. However, limitations exist and should be taken into account when interpreting our results and limits their generalization. The data stem from two studies with exploratory aims, not initially intended to address eating behaviours, limiting statistical power. Indeed the present study was not powered to detect changes in eating behaviours, hence, we cannot exclude the possibility that we might have not detected smaller effects of physical activity on cognitive restraint, uncontrolled eating and emotional eating, since we have a small number of participants (*n* = 27). Additionally, only 27 out of the 57 participants completed the TFEQ at both time points, possibly representing a subsample more motivated for example. However, we aimed to address this potential bias by conducting sensitivity analyses with imputed missing data. Furthermore, participants' responses to the TFEQ may be influenced by a desirability bias, given its focus on behaviours perceived as negative or stigmatized. Some items from the hunger factor in TFEQ‐51 overlap with uncontrolled eating in TFEQ‐R21 (e.g., Items 19, 24, 26, 34, 22 and 39). Similarly, items from TFEQ‐51 disinhibition factor align with TFEQ‐R21's emotional eating (e.g. Items 9 and 20), leading to double scoring and impacting outcomes. The lack of a control group complicates attributing observed changes in eating behaviours solely to physical activity. Data on participants' physical activity beyond the intervention were not gathered. Additionally, the fact that all participants did not engage in the same physical activity and some having received motivational techniques makes our sample somewhat heterogeneous.

## 6 Conclusion

Physical activity can influence disordered eating behaviours in people with psychotic disorders, possibly reducing uncontrolled and emotional eating without nutritional or psychological interventions. Further studies with larger samples and longer follow‐up periods are needed to grasp physical activity's role in disordered eating behaviours and explore its potential as a preventive measure against premature mortality from metabolic syndrome. Such insights could inform interventions to enhance physical health among individuals with psychotic disorders.

## Ethics Statement

The combined researchs studies were performed in accordance to the Declaration of Helsinki and were approved by the Ethics Committee of the research centre at the Centre Hospitalier de l'Université de Montréal (reference numbers 10.255 and 16.399; Dubois 2020).

## Consent

All participants provided written informed consent.

## Conflicts of Interest

The authors declare no conflicts of interest.

## Data Availability

The dataset used will be available upon reasonable request to the corresponding author.

## TFEQ's Harmonized

### 1

**Table:**

Factors	Items used	Items TFEQ‐R21	Items TFEQ‐51
Cognitive restraint^a^			
	1	1	6
	5	5	33
	11	11	28
	17	17	43
	18	18	48
	21	21	50
	4, 10, 14, 18, 21, 23, 30, 32, 35, 37, 38, 40, 42, 44, 46		4, 10, 14, 18, 21, 23, 30, 32, 35, 37, 38, 40, 42, 44, 46
Uncontrolled eating^b^			
	3	3	15
	6	6	19
	8	8	24
	9	9	26
	12	12	1
	13	13	34
	15	15	22
	19	19	49
	20	20	39
	2, 7, 11, 13, 16, 25, 31, 36, 45, 51		2, 7, 11, 13, 16, 25, 31, 36, 45, 51
Emotional eating^c^			
	2	2	9
	4	4	20
	10	10	27
	3, 5, 8, 12, 17, 29, 41, 47		3, 5, 8, 12, 17, 29, 41, 47

*Note*: Item 6 (TFEQ‐51) = Item 1 (TFEQ‐R21), Item 33 (TFEQ‐51) = Item 55 (TFEQ‐R21), Item 28 (TFEQ‐51) = Item 11 (TFEQ‐R21), Item 43 (TFEQ‐51) = Item 17 (TFEQ‐R21), Item 48 (TFEQ‐51) = Item 18 (TFEQ‐R21), Item 50 (TFEQ‐51) = Item 21 (TFEQ‐R21), Item 15 (TFEQ‐51) = Item 3 (TFEQ‐R21), Item 19 (TFEQ‐51) = Item 6 (TFEQ‐R21), Item 24 (TFEQ‐51) = Item 8 (TFEQ‐R21), Item 26 (TFEQ‐51) = Item 9 (TFEQ‐R21), Item 1 (TFEQ‐51) = Item 12 (TFEQ‐R21), Item 34 (TFEQ‐51) = Item 13 (TFEQ‐R21), Item 22 (TFEQ‐51) = Item 15 (TFEQ‐R21), Item 49 (TFEQ‐51) = Item 19 (TFEQ‐R21), Item 39 (TFEQ‐51) = Item 20 (TFEQ‐R21), Item 9 (TFEQ‐51) = Item 2 (TFEQ‐R21), Item 20 (TFEQ‐51) = Item 4 (TFEQ‐R21), Item 27 (TFEQ‐51) = Item 10 (TFEQ‐R21).

^a^ [(((Mean score of the items) * 6) − 6)/18] * 100.

^b^ [(((Mean score of the items) * 9) − 9)/27] * 100.

^c^ [(((Mean score of the items) * 6) − 6)/18] * 100.
